# The Prevalence of Congenital Heart Diseases in Syndromic Children at King Khalid National Guard Hospital from 2005 to 2016

**DOI:** 10.7759/cureus.7891

**Published:** 2020-04-29

**Authors:** Elaf M Abduljawad, Ahad AlHarthi, Samah A AlMatrafi, Mawaddah Hussain, Aiman Shawli, Rahaf Waggass

**Affiliations:** 1 Medicine, College of Medicine, King Saud Bin Abdulaziz University for Health Sciences, Jeddah, SAU; 2 Pediatrics, King Abdulaziz Medical City, Ministry of National Guard Health Affairs, Jeddah, SAU; 3 Pediatric Cardiology, King Saud Bin Abdulaziz University for Health Sciences, Jeddah, SAU

**Keywords:** congenital heart diseases, dysmorphic syndrome, down syndrome, digeorge syndrome, turner syndrome, atrial septal defect, patent ductus arteriosus, ventricular septal defect, children, syndromic children

## Abstract

Background

Congenital heart diseases (CHDs) are abnormalities that present in the heart since birth and are one of the leading causes of infant mortality in the world. CHDs are more common among children with dysmorphic syndromes. The current study aims to estimate the prevalence of many CHDs in different dysmorphic syndromes.

Methods

This was a retrospective chart review study conducted on all dysmorphic syndrome patients who attended genetic clinics at King Khalid National Guard Hospital in King Abdulaziz Medical City (KAMC), Jeddah, Saudi Arabia from 2005 to 2016. Dysmorphic pediatric patients less than 14 years old who had genetic testing to confirm their diagnosis were included in the study. Patients who did not have any previous echocardiography were excluded.

Results

A total of 212 individuals (47% males and 53% females) were included. Eighty-five percent of Down syndrome patients had CHDs, and the most common CHD was an atrial septal defect (ASD) (51%). In patients with Turner syndrome, 45% of them had CHDs, and bicuspid aortic valve (BAV) (40%) was the most common defect. In DiGeorge syndrome, 81% of patients had CHDs, and ventricular septal defect (VSD) (41%) was the most common. In Williams syndrome, 83% of patients had CHDs. All patients with Noonan, Edwards, CHARGE (coloboma, heart defects, atresia choanae (also known as choanal atresia), growth retardation, genital abnormalities, and ear abnormalities), and Rubinstein-Taybi syndromes were found to have CHDs. In Patau syndrome and Joubert syndrome, 50% of patients in each had CHDs. Patients with Prader Willi syndrome had normal findings in the echocardiogram.

Conclusion

The highest prevalence of CHDs was found in Down syndrome. This study has a significant impact on the future of managing and directing the resources to improve the quality of life for syndromic patients. Further studies are needed to confirm these findings and to increase the local data in the field of CHDs in Saudi Arabia among syndromic patients.

## Introduction

Congenital heart disease (CHD) is an abnormality that is present in the heart since birth which includes structural walls, valves, and major blood vessels of the heart, and it can be detected by echocardiography [1]. CHD affects the lives of approximately eight in 1,000 newborns daily and is one of the leading causes of infant mortality in the world [2]. In Saudi Arabia, the rate of CHD is high. Moreover, it affects approximately 11 in 1,000 newborns daily due to some factors, such as consanguinity, certain dysmorphic syndromes, and other significant risk factors [3]. There are many CHDs that can be detected in pediatric patients, like atrial septal defect (ASD), ventricular septal defect (VSD), atrioventricular septal defect (AVSD), patent ductus arteriosus (PDA), tetralogy of Fallot (TOF), bicuspid aortic valve (BAV), coarctation of the aorta (CoA), and others.

Dysmorphic syndrome is a syndrome that has characteristic facial or body features, such as up slanting eyes, low set ears, webbed neck, and others. Also, they are usually associated with cardiac or extracardiac manifestations. There are many dysmorphic syndromes which include Down, Turner, Patau, DiGeorge, Noonan, Edwards, Prader-Willi, Williams, Joubert, CHARGE (coloboma, heart defects, atresia choanae (also known as choanal atresia), growth retardation, genital abnormalities, and ear abnormalities), Rubinstein-Taybi, and other syndromes. In the study by Ko, it was shown that the previous dysmorphic syndromes have a higher prevalence of CHDs than the others [4]. Down syndrome (trisomy 21) is common in Saudi Arabia, and it is the most common genetic disorder that is associated with CHD, nearly half of them have CHD [5-6]. Moreover, it has characteristic dysmorphic features, such as upslanting eyes, short neck, and others [7]. Turner syndrome, that has an abnormal karyotype of xo 45, is a syndrome affecting females only and has distinct features, such as the webbed small neck, short stature, and other dysmorphic features [8]. The incidence of Turner syndrome worldwide is approximately one in 2,500 female births. Another dysmorphic syndrome is Patau syndrome (trisomy 13), which has a prevalence of one per 20,000 births worldwide [9]. DiGeorge syndrome, which may be caused by a microdeletion of a small segment in chromosome 22, has distinct facial features, such as small eyes, epicanthal folds, thin lips, and others [10]. Noonan syndrome, which has a prevalence of one in 2,500 births worldwide, can be diagnosed by detecting various gene mutations of PTPN11, SOS1, RADF1, and KRAS. Moreover, it has specific features, such as prominent eyes, short broad nose, and others [11]. Edwards syndrome is a common chromosomal disorder that has an additional chromosome in chromosome 18 with a prevalence of one in 2,500 lives worldwide [12]. Also, it is the second most common autosomal trisomy syndrome after Down syndrome. Prader-Willi syndrome has a disruption of genes in the proximal arm of chromosome 15 and is also the most common genetic cause of life-threatening childhood obesity [13]. Also, it has distinctive facial features, hypogonadism, intellectual disabilities, behavioral problems, and excessive eating [14]. Williams syndrome has a deletion of the 7q11.23 region in karyotype, and it has a prevalence of one in 7,500 births. Its dysmorphic features include star-like patterns in iris of eyes, small chin, and wide mouth [15]. Joubert syndrome is an autosomal recessive disorder with a prevalence of one in 80,000 births [16]. Also, it has a mutation in the INPP5E gene (613037) on the chromosome (9q34). It characterized by facial features of a large head and broad nose, ataxia, abnormal ocular movements, and psychomotor delay. CHARGE syndrome is another dysmorphic syndrome that has distinct ear malformation, genital malformation, and others. It has a prevalence of one in 10,000, and it is detected by a gene mutation in CHD7 chromosome 8 [17]. Rubinstein-Taybi syndrome has a prevalence of one in 125,000 births worldwide [18]. It characterized by dysmorphic features, such as broad thumb, toes, and clinodactyly. It is also diagnosed by detecting a mutation in CREB-binding protein on chromosome 16p13.

Due to the harmful impact of CHD, it is important to identify it in the early life of the patients with dysmorphic syndromes by directing the resources to the syndromes with a higher incidence of CHD. Our local data lacks statistics regarding this topic, so this research will help in identifying and directing the resources for the patients in our center, such as in providing early interventions, support groups, and perinatal screening.

There are two studies similar to our study in the way they discuss CHD. The first study was done in Saudi Arabia in 2012 by Al Aama et al. [5]. This study was a prospective study of 130 patients with Down syndrome seen in King Abdulaziz University Hospital, Jeddah from October 2007 to October 2011. Briefly, this study estimated CHDs and other major anomalies in patients with Down syndrome. In this study, CHDs were found in 86.8% of children with Down syndrome; ductus arteriosus (47.8%) was the most common CHD, followed by ASD (41.3%) [5]. The second study was a cross-sectional study with retrospective data collection which was conducted in Brazil by Mourato et al. [19]. This study included 138 children with Down syndrome. The study estimated that 81.2% of Down syndrome patients had CHDs, and ASD (51.8%) was the most common, followed by AVSD (46.4%), VSD (27.7%), then ToF (6.3%) [19]. Both studies discussed CHDs specifically in patients with Down syndrome. However, our study aim is to estimate the prevalence of many CHDs in different dysmorphic syndromes, which then gives us a better way of approaching the patients at King Khaled Hospital in Jeddah. 

## Materials and methods

Study design, setting, and participants 

This was a retrospective chart review study that was conducted at King Khalid Hospital for National Guard in King Abdulaziz Medical City (KAMC), Jeddah, Saudi Arabia on 212 syndromic patients who were being followed up at pediatric genetic clinics from the period from 2005 to 2016. King Khalid Hospital for National Guard in KAMC is one of the tertiary care centers in Jeddah where around 300 pediatric patients have been diagnosed with dysmorphic syndromes every year. Furthermore, it is an excellent referral center that accepts patients from all major cities in the western region in Saudi Arabia. The genetic clinics at King Khalid Hospital for National Guard in KAMC manage pediatric patients with genetic diseases, including dysmorphic syndromes, and provide comprehensive multidisciplinary services, such as parental testing for genetic syndromes and anticipatory clinical guidance, as well as testing for congenital cardiac defects. Eligible patients had to be pediatric patients (below the age of 14 years old) who had a diagnosis of a dysmorphic syndrome and had a previous echocardiogram that was evaluated by a cardiologist. To meet the criteria of diagnosing a dysmorphic syndrome, patients had to be diagnosed based on chromosomal analysis or molecular tests, not merely on clinical suspicion. The 11 dysmorphic syndromes included in the study were Down syndrome, Turner syndrome, Patau syndrome (trisomy 13), DiGeorge syndrome, Noonan syndrome, Edwards syndrome, Prader-Willi syndrome, Williams syndrome, Joubert syndrome, CHARGE syndrome, Rubinstein-Taybi syndrome, and others. In echocardiography results, all congenital heart defects were included in the study, except patent foramen ovale (PFO), which is considered a normal CHD. The abnormal CHDs included ASD, VSD, AVSD, PDA, TOF, BAV, CoA, and others. Patients who did not undergo echocardiography were excluded from this study. 

Material and data analysis

The sample size was estimated at the 95% confidence level (CI) with an estimated 61% response distribution and a margin of error of ± 5%. A non-probability consecutive sampling technique was used, and it covered the period from 2005 to 2016. Furthermore, this was a secondary data collection where the data were collected by the coauthors of this study from the medical records, cytogenic lab files, BestCare software (Shenzhen Bestcare Biomedical Co., Ltd., Shenzhen, China), and Xcelera software (Philips, Amsterdam, The Netherlands) using a predesigned data collection sheet, which included patients’ gender, type of syndrome, and results of echocardiography; codes were used to ease the process of data entry. The data entry was done by using Microsoft® Excel sheets (Microsoft® Corp., Redmond, WA). The data was then transformed into the Statistical Package for Social Sciences (SPSS) (SPSS Statistics, Chicago, IL) PASW (Predictive Analytics Software) statistics version 18 to carry out data analysis. For categorical variables (which were gender, syndrome, and CHDs), frequencies and percentages were reported. Chi-square test (or Fisher exact tests for cells less than 5) were used to see the difference of frequencies of different CHDs in each syndrome, and a p-value of less than 0.05 was significant.

Ethical consideration

Only patients’ medical records were reviewed (informed consent was not required). Patients’ files were approached in the medical records department. Patient privacy and confidentiality were assured, no identities were collected, and all data was kept in a secure place within National Guard Health Affairs (NGHA) premises in both hard and soft copies. Moreover, all procedures performed in this study were in accordance with the ethical standards of the institutional research committee and with the 1964 Helsinki declaration and its later amendments. The study met all institutional ethical board requirements by King Abdullah International Medical Research Center (KAIMRC).

## Results

**Figure 1:**
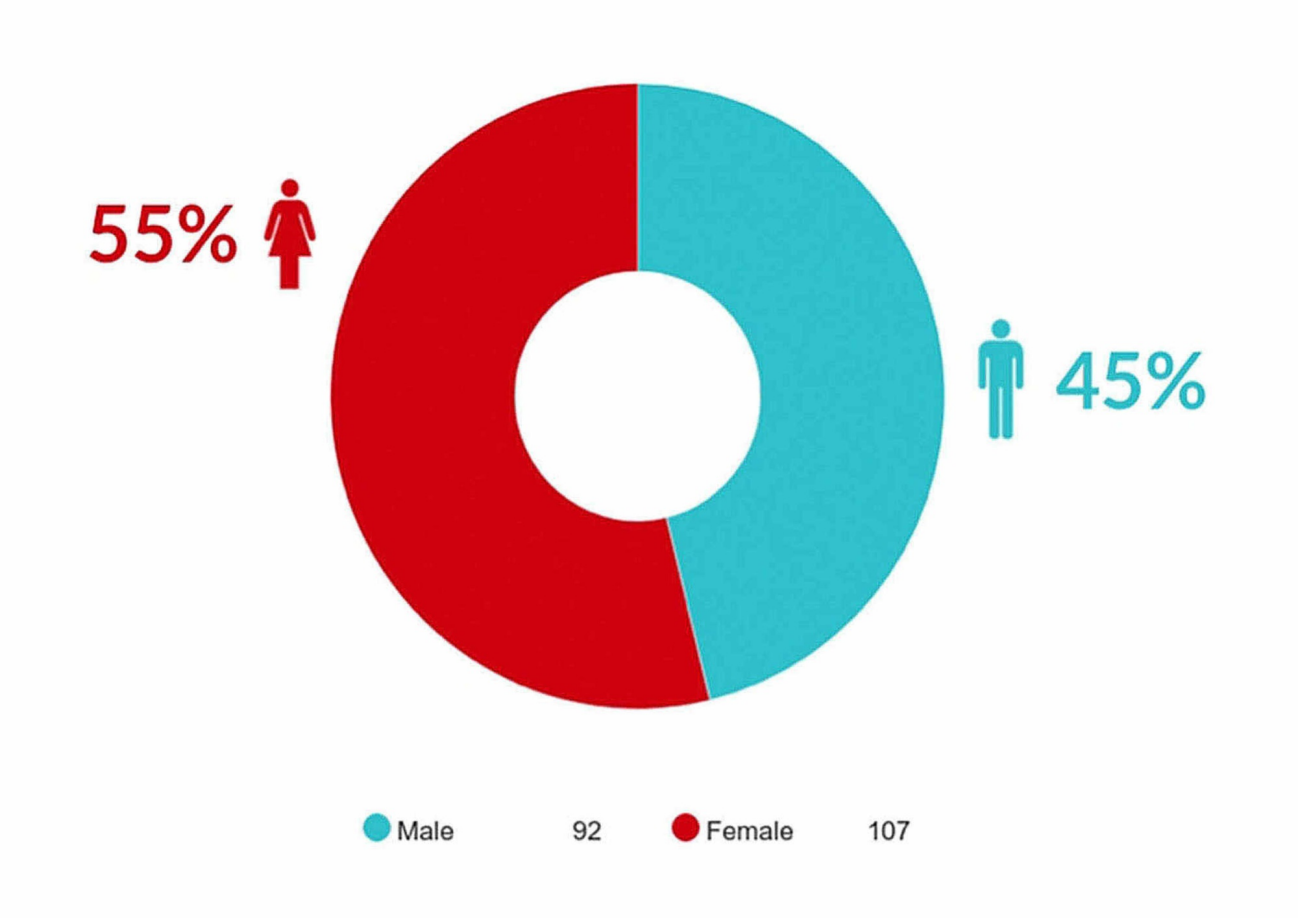
Demographic Characteristics (Gender)

**Figure 2:**
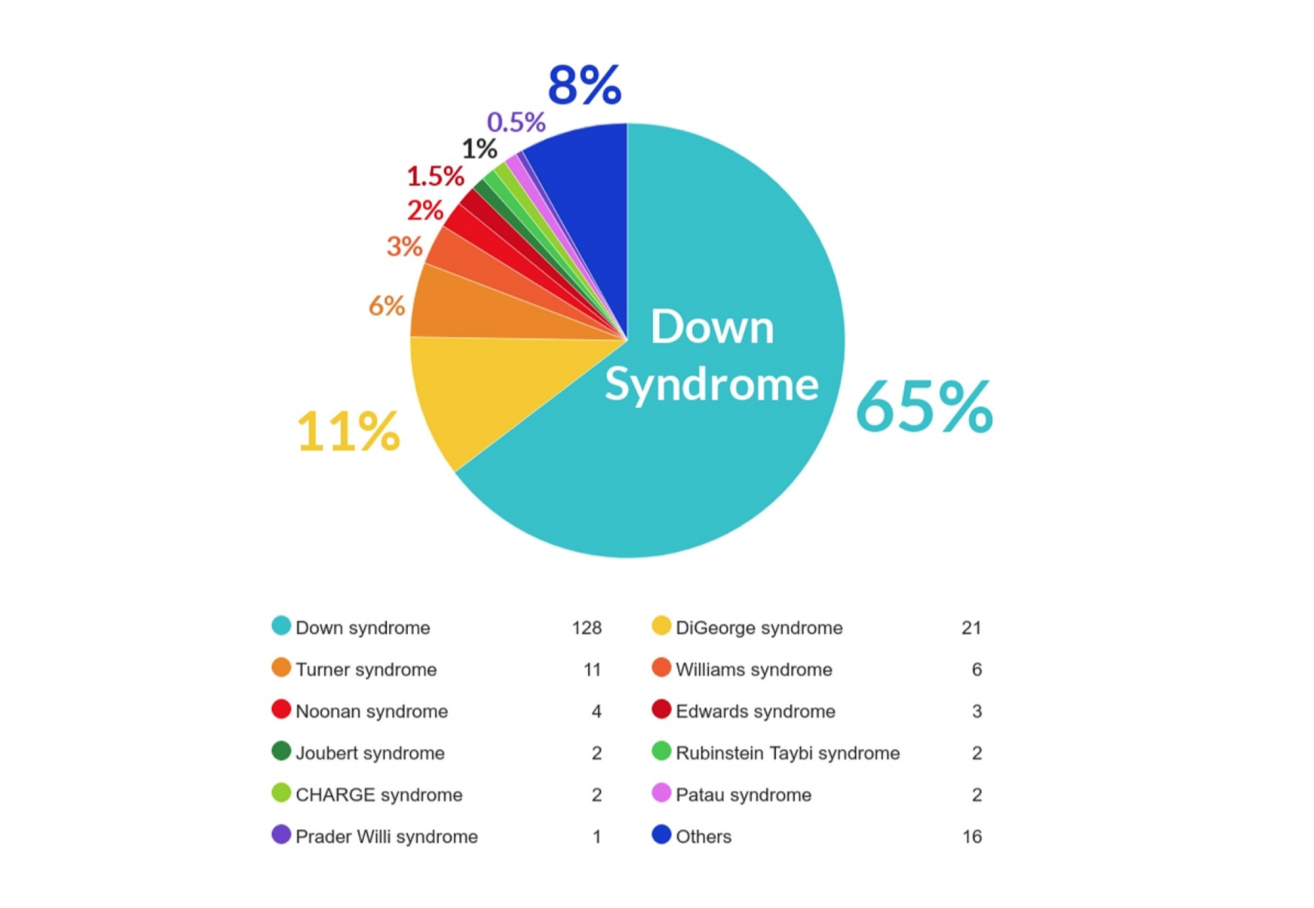
Genetic Syndromes

**Figure 3:**
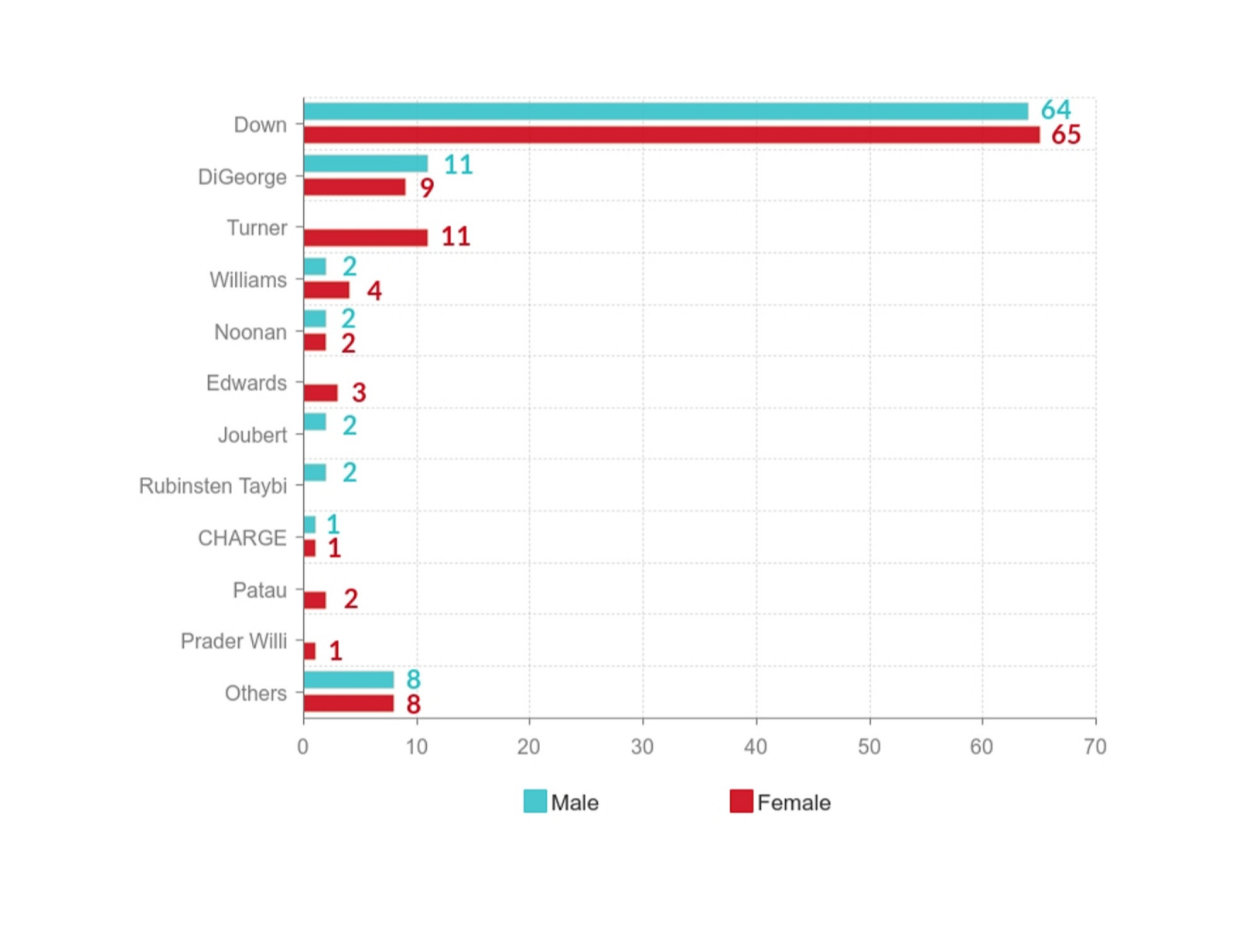
Relationship Between Gender and Each Genetic Syndrome

Data were collected on a total of 212 subjects, but 14 out of the 212 patients who had patent foramen ovale (PFO) were excluded. One hundred and ninety-eight subjects met the entry criteria (n = 198), including 92 (45%) males and 107 (55%) females (M: F = 1: 1.2) (Figure 1). The karyotype test showed that 129 subjects (65%) had Down syndrome (64 of whom were males and 65 were females). Twenty-one subjects (10.6%) had DiGeorge syndrome (11 of whom were males and 10 were females). Eleven subjects (5.6%) had Turner syndrome (all females). Six subjects (3%) had Williams (two were males and four were females). Four subjects (2%) had Noonan syndrome (two of whom were males and two were females). Three subjects (1.5%) had Edwards syndrome (all were females). Two subjects (1%) were assigned into each of the following syndromes: Joubert syndrome (all males), Rubinstein-Taybi syndrome (all were males), CHARGE syndrome (one male and one female), and Patau syndrome (all were females). Only one female subject (0.5%) had Prader Willi syndrome. There were 16 patients (8.1%) with other syndromes which included Smith Limo Opitz syndrome, deletion of ch13, Alagille syndrome, Sanjad Sakati syndrome, Pierre Robin sequence, Marfan syndrome, mosaic trisomy 8, Coffin-Siris syndrome, fetal akinesia sequence, Robert syndrome, Raine syndrome, Eisenmenger syndrome, Peters Plus syndrome, Vater association 5, Wolf Hirschhorn syndrome (4q deletion), deletion of chromosome 7q, and Raine syndrome as reported in Figure 2. The relationship between gender and each genetic syndrome is shown in Figure 3.

**Figure 4:**
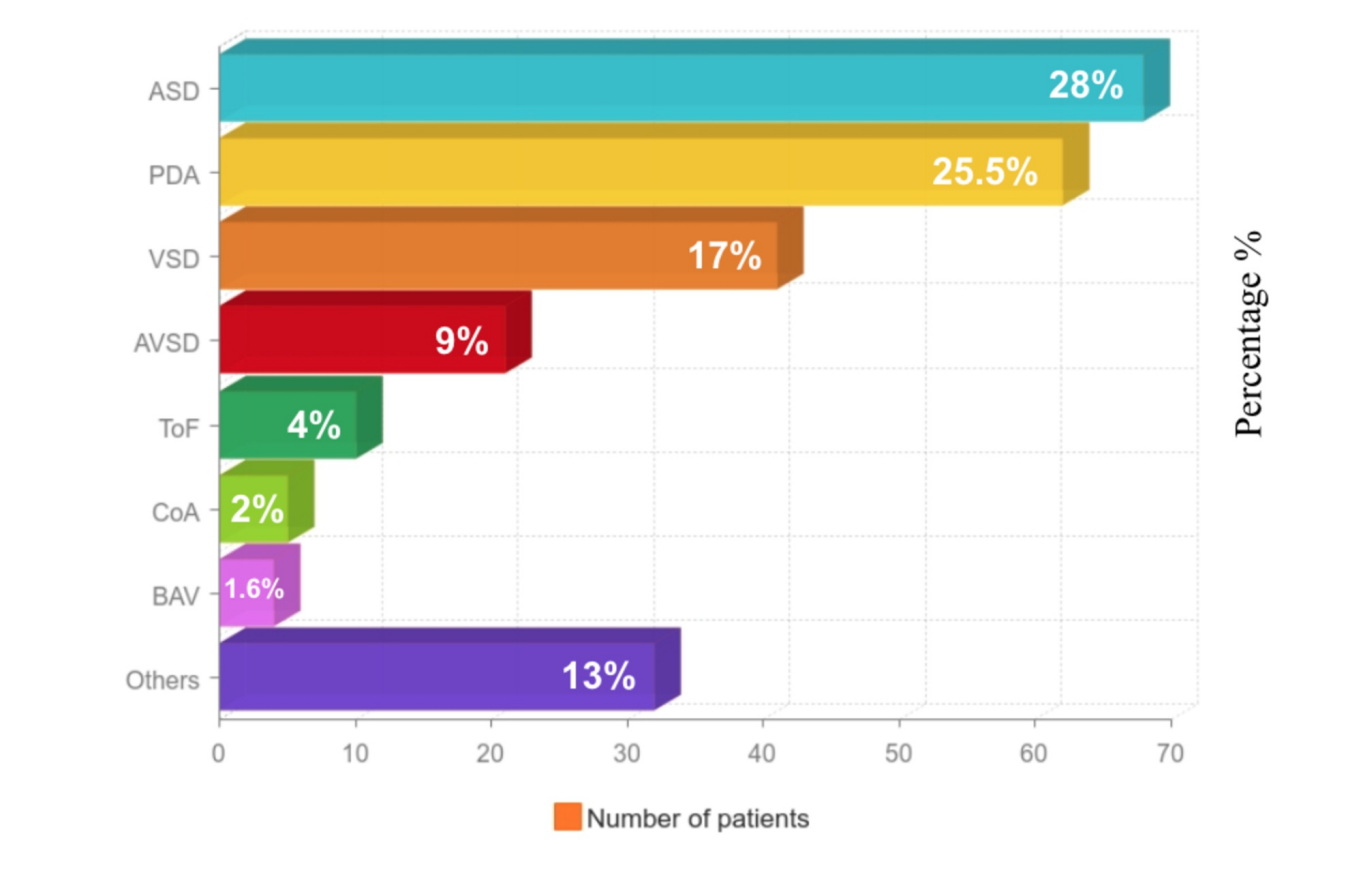
Congenital Heart Diseases ASD: atrial septal defect; AVSD: arterial vascular septal defect; BAV: bicuspid aortic valve; CoA: coarctation of the aorta; PDA: patent ductus arteriosus; ToF: tetralogy of Fallot; VSD: ventricular septal defect

Congenital heart diseases (CHDs) were discovered in 159 of the patients (80.3%). The major CHDs are shown in Figure 4. Thirty-nine of the children had normal findings. The total of all CHDs presented in the dysmorphic patients who had abnormal findings in the echocardiogram were 243 in total in which patients had single or multiple CHDs. We found that 68 patients (28%) had an atrial septal defect (ASD), 62 patients (25.5%) had patent ductus arteriosus (PDA), 41 patients (16.9%) had a ventricular septal defect (VSD), 21 patients (8.6%) arterial vascular septal defect (AVSD), 10 patients (4.1%) tetralogy of Fallot (ToF), five patients (2.1%) had coarctation of the aorta (CoA), four patients (1.6%) had bicuspid aortic valve (BAV), and 32 patients (13.2%) had other CHDs which included tricuspid regurgitation, pulmonary artery stenosis, mitral regurgitation, thickened mitral valve, aneurysmal atrial septum, aortic regurgitation, mitral valve prolapse, dilated left coronary artery, supra-aortic stenosis, narrow aortic arch, atrioventricular valve regurgitation, dilated pulmonary valve, pulmonary artery stenosis, atrial aneurysm, vertical vein to superior vena cava, and tortuous aortic arch with distal narrowing. There was no statistical significance in the difference between males and females in all of the dysmorphic syndromes and the CHDs.

**Figure 5:**
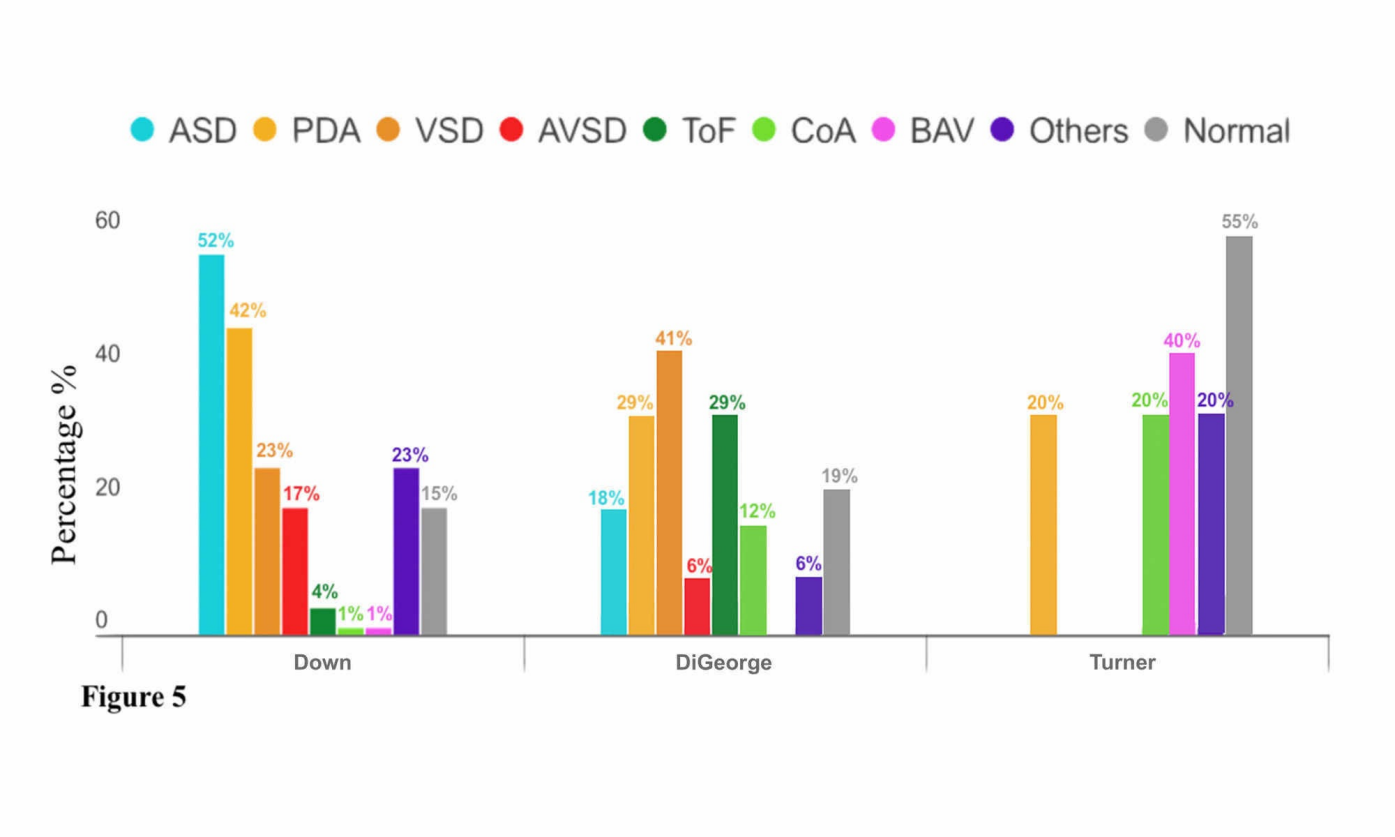
Relationship Between Congenital Heart Diseases and Genetic Syndromes (Down, DiGeorge, and Turner Syndromes) ASD: atrial septal defect; AVSD: arterial vascular septal defect; BAV: bicuspid aortic valve; CoA: coarctation of the aorta; PDA: patent ductus arteriosus; ToF: tetralogy of Fallot; VSD: ventricular septal defect

In different dysmorphic syndromes, CHDs were reported with various numbers. There were 129 subjects diagnosed with Down syndrome; 19 (14.8%) of them had normal echo findings. On the other hand, the other 110 patients (P-value = 0.026) had single or multiple abnormal echo findings. There were 178 anomalies in total, and they are reported in Figure 5. The anomalies included 57 patients (51.81%) who had ASD, 46 patients (41.81%) had PDA, 25 patients (22.72%) had VSD, 19 patients (17.27%) had AVSD, four patients (3.63%) had ToF, one patient (0.90%) had BAV, one patient (0.90%) had CoA, and 25 patients (22.72%) had other CHDs.

There were 21 subjects diagnosed with DiGeorge syndrome, four (19%) of which had normal echo findings. On the other hand, the other 17 patients (P-value = 0.93) had 24 single or multiple abnormal echo findings, which included three patients (17.64%) who had ASD, five patients (29.41%) had PDA, seven patients (41.17%) had VSD, one patient (5.88%) had AVSD, five patients (29.41%) had ToF, two patients (11.76%) had CoA, and one patient (5.88%) had other CHDs (Figure 5).

There were 11 subjects diagnosed with Turner syndrome, six (54.54%) of which had normal echo findings. On the other hand, the other five patients (P-value = 0.01) had single abnormal echo findings as reported in Figure 5, which included one patient (20%) who had PDA, two patients (40%) had BAV, one patient (20%) had CoA, and one patient (20%) had other CHDs.

**Figure 6:**
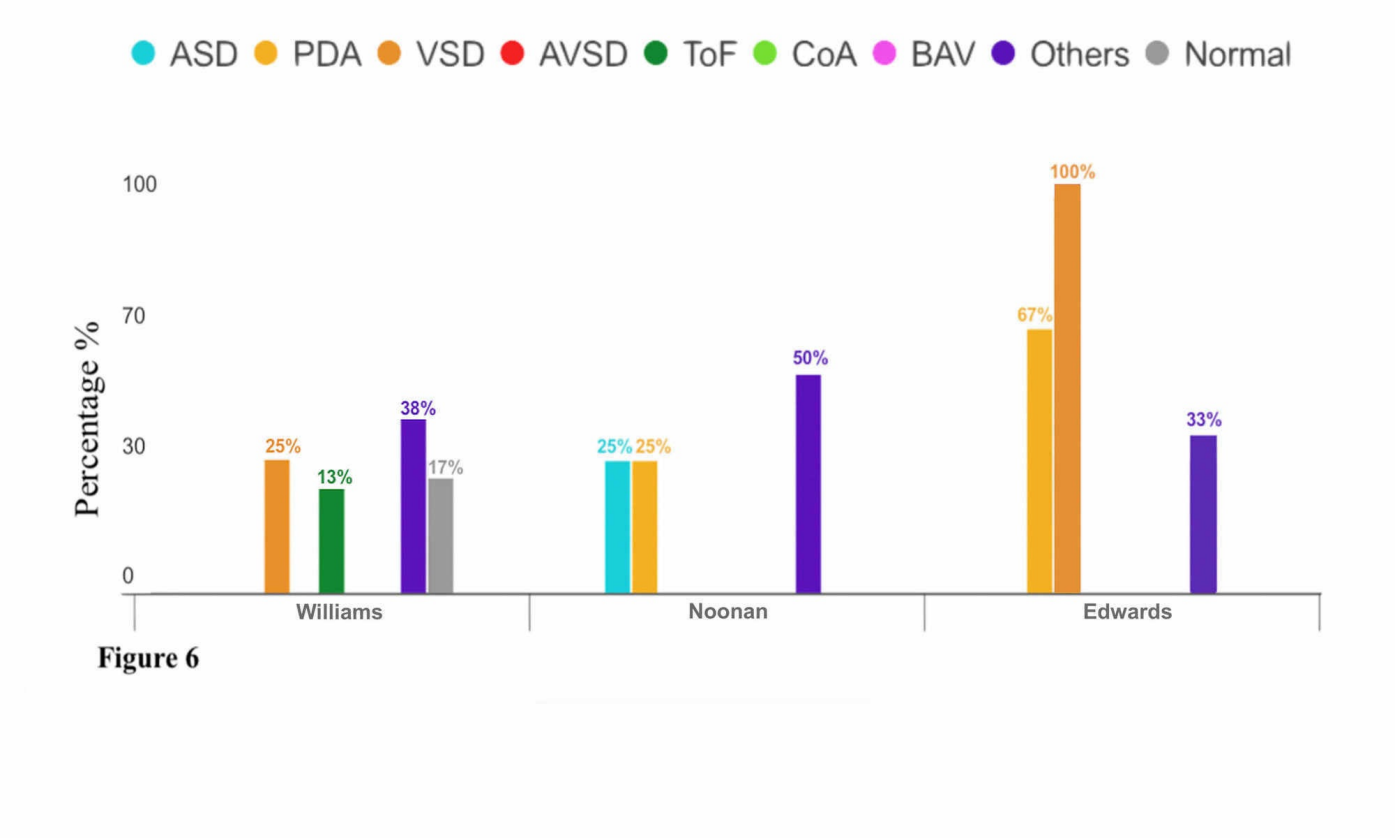
Relationship Between Congenital Heart Diseases and Genetic Syndromes (Williams, Noonan, and Edwards Syndromes)

There were six subjects diagnosed with Williams syndrome, one (16.7%) of which had normal echo findings. On the other hand, the other five patients (P-value = 0.91) had six single or multiple abnormal echo findings as reported in Figure 6, which included two patients (25%) who had VSD, one patient (12.5%) had ToF, and three patients (37.5%) had other CHDs.

There were four subjects diagnosed with Noonan syndrome. All patients (P-value = 0.28) had four single abnormal echo findings as reported in Figure 6, which included one patient (25%) who had ASD, one patient (25%) had PDA, and two patients (50%) had other CHDs.

There were three subjects diagnosed with Edwards syndrome. All patients (P-value = 0.40) had six single or multiple abnormal echo findings as reported in Figure 6, which included two patients (66.66%) who had PDA, three patients (100%) had VSD, and one patient (33.33%) had other CHDs.

**Figure 7:**
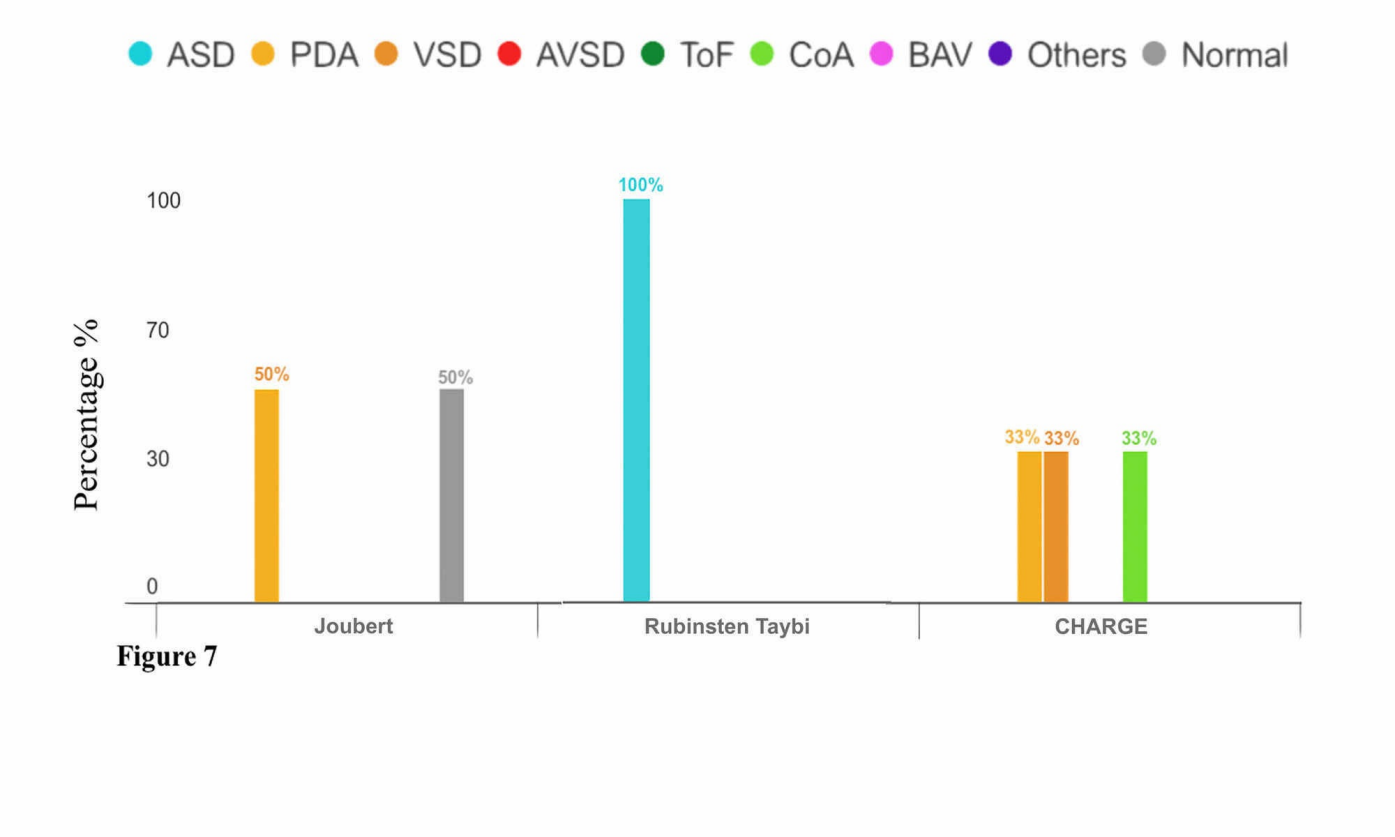
Relationship Between Congenital Heart Diseases and Genetic Syndromes (Joubert, Rubinstein-Taybi, and CHARGE Syndromes) CHARGE: coloboma, heart defects, atresia choanae (also known as choanal atresia), growth retardation, genital abnormalities, and ear abnormalities

There were two subjects diagnosed with Joubert syndrome, one of which (50%) had a normal echo finding. The other one (P-value = 0.50) had PDA as reported in Figure 7.

There were two subjects diagnosed with Rubinstein-Taybi syndrome, neither of which had normal echo findings. On the other hand, all patients (P-value = 0.36) had ASD as reported in Figure 7.

There were two subjects diagnosed with CHARGE syndrome, neither of which had normal echo findings. All patients (P-value = 0.50) had three single or multiple abnormal echo findings as reported in Figure 7, which included one patient (33.3%) who had CoA, and the other patient who had VSD (33.3%) and PDA (33.3%).

**Figure 8:**
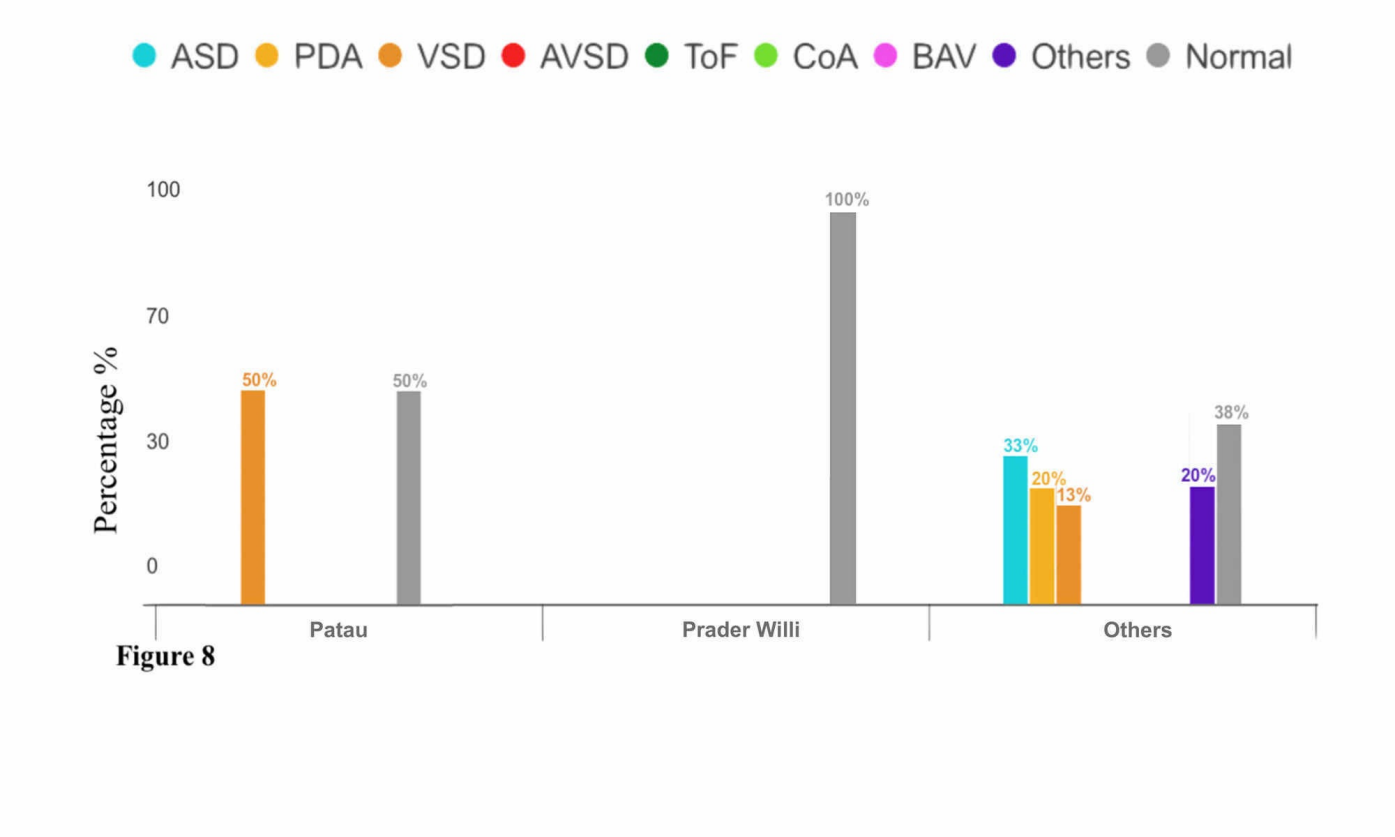
Relationship Between Congenital Heart Diseases and Genetic Syndromes (Patau, Prader Willi, and Other Syndromes)

There were two subjects diagnosed with Patau syndrome, one of which (50%) had a normal echo finding. The other subject (P-value = 0.24) had VSD, as reported in Figure 8. One patient was diagnosed with Prader Willi syndrome and had normal echo finding as reported in Figure 8. There were 16 subjects diagnosed with other genetic syndromes, six (37.5%) of which had normal echo findings. On the other hand, there were 13 cardiac defects in total in the other 10 patients including five patients (33.33%) who had ASD, three patients (20%) had PDA, two patients (13.33%) had VSD, and three patients (20%) had other CHDs as reported in Figure 8.

Table 1 shows a summary of the percentages of specific CHDs in each syndrome and the most common type in each. In Table 2, the frequency of associated CHDs in various dysmorphic syndromes in the current study was compared to a group of national and international figures which was calculated using the Chi-square test and will be explained in detail in the Discussion section.

**Table 1:** Summary of the Relationship between Congenital Heart Diseases and Genetic Syndromes ASD: atrial septal defect; AVSD: arterial vascular septal defect; BAV: bicuspid aortic valve; CHARGE: coloboma, heart defects, atresia choanae (also known as choanal atresia), growth retardation, genital abnormalities, and ear abnormalities; CoA: coarctation of the aorta; #: number; PDA: patent ductus arteriosus; pt: patients; ToF: tetralogy of Fallot; VSD: ventricular septal defect

Type of syndrome	# of pt	ASD	PDA	VSD	AVSD	ToF	CoA	BAV	Others	Normal
Down syndrome	129	57 (51.81%)	46 (41.81%)	25 (22.72%)	19 (17.27%)	4 (3.63%)	1 (0.9%)	1 (0.9%)	25 (22.72%)	19 (14.72%)
DiGeorge syndrome	21	3 (17.64%)	5 (29.41%)	7 (41.17%)	1 (5.88%)	5 (29.41%)	2 (11.76%)	0	1 (5.88%)	4 (19%)
Turner syndrome	11	0	1 (20%)	0	0	0	1 (20%)	2 (40%)	1 (20%)	6 (54.54%)
Williams syndrome	6	0	0	2 (25%)	0	1 (12.5%)	0	0	3 (37.5%)	1 (16.7%)
Noonan syndrome	4	1 (25%)	1 (25%)	0	0	0	0	0	2 (50%)	0
Edwards syndrome	3	0	2 (66.66%)	3 (100%)	0	0	0	0	1 (33.33%)	0
Joubert syndrome	2	0	1 (50%)	0	0	0	0	0	0	1 (50%)
Rubinstein-Taybi syndrome	2	2 (100%)	0	0	0	0	0	0	0	0
CHARGE syndrome	2	0	1 (33.3%)	1 (33.3%)	0	0	1 (33.3%)	0	0	0
Patau syndrome	2	0	0	1 (50%)	0	0	0	0	0	1 (50%)
Prader Willi syndrome	1	0	0	0	0	0	0	0	0	1 (100%)
Other syndromes	16	5 (33.33%)	3 (20%)	2 (13.33%)	0	0	0	0	3 (20%)	6 (37.5%)

**Table 2:** Comparison Between the Results of Our Study with National and International Studies CHARGE: coloboma, heart defects, atresia choanae (also known as choanal atresia), growth retardation, genital abnormalities, and ear abnormalities; N/A: not available

Type of syndrome	P-value	# of Patients	Other Studies	n
Down syndrome	85% (p = 0.026)	129	Digilio et al. [10]; 86.6%	130
DiGeorge syndrome	81% (p = 0.935)	21	Marino et al. [11]; 75%	49,458
Turner syndrome	50% (p = 0.010)	11	Cereda et al. [12]; 23%	594
Williams syndrome	83.3% (p = 0.912)	6	Marcus et al. [13]; 83%	113
Noonan syndrome	100% (p = 0.283)	4	Torrado et al. [14]; 50-80%	40
Edwards syndrome	100% (p = 0.408)	3	Strømme [15]; 94%	134
Joubert syndrome	50% (p = 0.501)	2	Stevens et al. [18]; 1 case report	N/A
Rubinstein Taybi syndrome	100% (p = 0.366)	2	Brancati et al. [16]; 24-38%	134
Charge syndrome	100% (p = 0.500)	2	Mourato et al. [19]; 75-85%	59
Patau syndrome	50% (p = 0.246)	2	Blake et al. [17]; 79%	23
Prader Willi syndrome	0% (p = 0.246)	1	Morsy et al. [20]; 47%	9

## Discussion

This study explored the prevalence of CHDs in different dysmorphic children at King Khaled Hospital for the National Guard, Jeddah. One hundred and ninety-eight patients (129 with Down syndrome, 21 with DiGeorge syndrome, 11 with Turner syndrome, six with Williams syndrome, four with Noonan syndrome, three with Edwards syndrome, two with Joubert syndrome, two with Rubinstein-Taybi syndrome, two with CHARGE syndrome, two with Patau syndrome, one with Prader-Willi syndrome, and 16 with other syndromes) were studied. Among these 198 patients, we found that the prevalence of CHDs differed from one syndrome to another, and we found some similarities and differences in previous studies [19-30].

Down syndrome (trisomy 21) is common in Saudi Arabia, and it is the most common genetic disorder that is associated with CHD. In Down syndrome, we found 85% of the subjects had CHDs, which is higher than local and international studies [19-21]. This is similar to the prevalence of CHD in Down syndrome previously stated by Al-Aama et al. Moreover, the Al-Aama et al. study showed that the most common defect among Down syndrome patients was PDA (47%). However, the most common CHD among Down syndrome patients in our study was ASD (41.81%).

DiGeorge is a syndrome that may be caused by a microdeletion of a small segment in chromosome 22. In patients with DiGeorge syndrome, our study found that 81% of them had CHDs, and this is very close to four studies done before and their results ranged between 75% - 86% [22-25]. Among these four studies, some showed TOF as the most common defect in patients with DiGeorge syndrome. However, others considered VSD (41.17%), which was similar to our research.

Turner syndrome, which has an abnormal karyotype of XO 45, is a syndrome affecting females only. In regard to Turner syndrome, our study found that 50% of patients had CHDs, which is higher than previous studies that ranged between 23% - 33% [26-27]. Similar to the previous studies, we found that BAV was the most common CHD which presented in 40% of patients with Turner syndrome, followed by CoA (20%), and this might be due to genetic reasons.

Williams syndrome has a deletion of the 7q11.23 region in the karyotype. We found that 83% of the patients with Williams syndrome had CHDs. This is similar to the prevalence of CHD in Williams syndrome previously stated by Collins [28].

Noonan is a syndrome that can be diagnosed by detecting various gene mutations of PTPN11, SOS1, RADF1, and KRAS. Previous studies showed that CHDs presented in 50% - 80% of patients with Noonan syndrome [29]. In comparison to our study, we had five patients with Noonan syndrome and all of them had CHDs.

Both our study and a previous study found that VSD was the most common defect among patients with Edwards syndrome [30]. Joubert syndrome, which is an autosomal recessive disorder, has a mutation in the INPP5E gene (613037) on chromosome 9q34. There is no previous study showing the prevalence of CHD in Joubert syndrome. In our study, we found that 50% of Joubert syndrome patients had CHDs and the most commonly presented with VSD.

We had two patients with Patau syndrome, and one of them had VSD. However, in the Polli et al. study, a heart defect was verified in 79% of cases with Patau syndrome, and ASD was the most common (53%). Moreover, we found that 100% of the Edwards, Rubinstein-Taybi, and CHARGE syndrome cases presented with CHDs.

This study has a few limitations. It was conducted at a single tertiary care center in central Saudi Arabia, and yet, the sample size was small. Secondly, the retrospective design was based on reviewing medical records, in turn, making the data more prone to be missed due to human error and inadequate documentation. The multicenter study would have a substantial effect on the sample size, as well as including diversifying patient backgrounds.

## Conclusions

In conclusion, we conducted this study to estimate the prevalence of CHDs among dysmorphic syndromic children at King Khalid National Guard Hospital in King Abdulaziz Medical City (KAMC), Jeddah, Saudi Arabia. It showed that the highest prevalence of CHDs was found in Down syndrome. This study has a significant impact on the future of managing and directing the resources to improve the quality of life for syndromic patients. Further studies are needed to confirm these findings and to increase the local data in the field of CHDs in SA among syndromic patients.
